# Prevalence and distribution of beta-lactamase coding genes in third-generation cephalosporin-resistant Enterobacteriaceae from bloodstream infections in Cambodia

**DOI:** 10.1007/s10096-015-2350-9

**Published:** 2015-02-26

**Authors:** E. R. Vlieghe, T.-D. Huang, T. Phe, P. Bogaerts, C. Berhin, B. De Smet, W. E. Peetermans, J. A. Jacobs, Y. Glupczynski

**Affiliations:** 1 Department of Clinical Sciences, Institute of Tropical Medicine, Nationalestraat 155, 2000 Antwerp, Belgium; 2 National Reference Laboratory for Resistance Monitoring of Gram-negative Bacteria, CHU Dinant-Godinne UCL, Avenue G. Thérasse 1, 5530 Yvoir, Belgium; 3 Sihanouk Hospital Centre of HOPE, Phnom Penh, Cambodia; 4 Department of Internal Medicine, University Hospital, KU Leuven—University of Leuven, Leuven, Belgium; 5 Department of Microbiology and Immunology, KU Leuven—University of Leuven, Leuven, Belgium

## Abstract

Resistance to third-generation cephalosporins in Gram-negative bacteria is emerging in Asia. We report the prevalence and distribution of extended-spectrum beta-lactamase (ESBL), AmpC beta-lactamase and carbapenemase-coding genes in cefotaxime-resistant Enterobacteriaceae isolates from bloodstream infections (BSI) in Cambodia. All Enterobacteriaceae isolated from BSI in adult patients at Sihanouk Hospital Centre of HOPE, Phnom Penh, Cambodia (2007–2010) were assessed. Antimicrobial susceptibility testing was carried out by disc diffusion and MicroScan according to Clinical and Laboratory Standards Institute (CLSI) guidelines. Screening for ESBL, plasmidic AmpC and carbapenemase-coding genes was performed by multiplex polymerase chain reaction (PCR) sequencing assays. Identification of the ST131 clone was performed in all CTX-M-positive *Escherichia coli*, using PCR targeting the *papB* gene. Out of 183 Enterobacteriaceae, 91 (49.7 %) isolates (84 BSI episodes) were cefotaxime-resistant: *E. coli* (*n* = 68), *Klebsiella pneumoniae* (*n* = 17) and *Enterobacter* spp. (*n* = 6). Most episodes were community-acquired (66/84; 78.3 %). ESBLs were present in 89/91 (97.8 %) cefotaxime-resistant isolates: 86 (96.6 %) were CTX-M, mainly CTX-M-15 (*n* = 41) and CTX-M-14 (*n* = 21). CTX-M of group 1 were frequently associated with TEM and/or OXA-1/30 coding genes and with phenotypic combined resistance to ciprofloxacin, sulphamethoxazole–trimethoprim and gentamicin (39/50, 78.0 %). Plasmidic AmpC (CMY-2 and DHA-1 types) were found alone (*n* = 2) or in combination with ESBL (*n* = 4). Eighteen *E. coli* isolates were identified as B2-ST131-O25B: 11 (61.1 %) carried CTX-M-14. No carbapenemase-coding genes were detected. ESBL among Enterobacteriaceae from BSI in Cambodia is common, mainly associated with CTX-M-15 and CTX-M-14. These findings warrant urgent action for the containment of antibiotic resistance in Cambodia.

## Introduction

Resistance to third-generation cephalosporins in Gram-negative bacteria has been associated with increased healthcare costs and higher rates of inappropriate therapy and mortality [1, 2]. The predominant underlying mechanism is the presence of plasmid-mediated extended-spectrum beta-lactamase (ESBL), mainly of the CTX-M, SHV or TEM types. Over the past decade, CTX-M has become the most prevalent ESBL type worldwide [3].

High rates of ESBL positivity among *Escherichia coli*, *Klebsiella* species and other Enterobacteriaceae have been described across Asia, in both hospital as well as community settings [4]. CTX-M, particularly CTX-M-15 and CTX-M-14. have been reported as the most common ESBL types in the Indian subcontinent, Southeast and East Asia, although patterns may differ locally [5]. Common risk factors for the acquisition of ESBL-producing strains in Southeast Asia include previous exposure to antibiotics (particularly cephalosporins and fluoroquinolones) and recent (i.e. <3 months) or actual hospitalisation [6, 7].

For Cambodia, data on prevalence, types and mechanisms of ESBL are very scarce. In a prospective cohort study of 93 patients with urinary tract infection, Ruppé and coworkers reported the presence of ESBL (all of the CTX-M type) in 37.7 % of *E. coli* from urine [8].

In a recent study in Cambodian hospitalised adults, we described the predominance of Gram-negative bacteria (mostly *E. coli*) in bloodstream infections (BSI), with a high resistance rate (50 %) to third-generation cephalosporins in Enterobacteriaceae and frequent association of co-resistance to fluoroquinolones, sulphamethoxazole–trimethoprim (SMX-TMP) and gentamicin [9]. Here, we report the mechanisms underlying these complex resistance patterns in Enterobacteriaceae isolates from Cambodian adult patients with BSI.

## Materials and methods

### Microbiological data

Sihanouk Hospital Centre of HOPE (SHCH) is a 40-bed non-governmental organisation (NGO) hospital for adults in Phnom Penh, Cambodia. It provides free care for the poor, with specific focus on patients with the human immunodeficiency virus (HIV) and the chronically ill. Microbiological facilities were installed in 2005, along with a local capacity-building programme focusing on the diagnosis and management of infectious diseases at the hospital level. In July 2007, a prospective study was started in patients presenting with presumed BSI.

Between 2007 and 2010, blood cultures with registration of demographic and clinical data were systematically obtained for all adult patients presenting with signs of systemic inflammatory response syndrome (SIRS) [10]. Venous blood (2 × 10 ml) was cultured in homemade brain heart infusion broth bottles (Bio-Rad, Berkeley, CA, USA) (July 2007–March 2009) and from April 2009 onwards in BacT/ALERT culture bottles (bioMérieux, Marcy l’Etoile, France). Blood cultures were incubated for 7 days at 35 °C and were monitored daily for growth. As part of standard patient care, isolates were identified by conventional biochemical tests and assessed for antibiotic susceptibility by disc diffusion. Isolates were stored at −70 °C on porous beads in cryopreservative (Microbank, Pro-Lab Diagnostics, Richmond Hill, Canada), and were shipped in batches to the Institute of Tropical Medicine (ITM), Antwerp (September 2010 and January 2011), where identification and susceptibility testing were verified with MicroScan (Siemens, Germany) according to Clinical and Laboratory Standards Institute (CLSI) guidelines (M100-S22) [11]. Isolates displaying intermediate susceptibility were considered resistant. All cefotaxime-resistant isolates from this collection were then referred to the National Reference Laboratory (Centre Hospitalier Universitaire Dinant-Godinne UCL) for confirmation of ESBL expression and genotypic characterisation of the isolates.

Screening for genes encoding conventional ESBL (CTX-M of groups 1, 2 and 9, SHV, TEM), minor ESBL (VEB, PER, BEL, GES), pAmpC and carbapenemase (VIM, IMP, NDM, OXA-48, KPC) was done by an end-point multiplex polymerase chain reaction (PCR) assay using a set of four validated (ISO 15189 standard) assays [12, 13], followed by sequencing of all the CTX-M genes detected. Sequence homology was determined by the BLASTX search tool using a non-redundant protein sequences’ database and comparison with the Lahey database (http://www.lahey.org/studies/).

Identification of the ST131 clone was performed in CTX-M-positive *E. coli*, using PCR targeting the *papB* gene [14].

### Clinical data and definitions

Basic clinical and epidemiological data were collected in all patients during the study through the laboratory request form. Infections were considered ‘nosocomial’ if they occurred more than two days after hospitalisation and ‘community-acquired’ if starting before or during the two first days of hospitalisation.

### Statistical analysis

Differences in proportions were assessed using Fisher’s exact test and considered statistically significant at *p*-values < 0.05. Data were analysed using Stata version 10.2 (StataCorp, College Station, TX, USA) and Excel 2003 (Microsoft Corporation, Redmond, WA, USA).

### Ethical considerations

Ethical approval was granted from the review boards at the Institute of Tropical Medicine, Antwerp, the University Hospital Antwerp and the National Ethics Committee for Health Research, Phnom Penh, Cambodia. Patients were identified with a unique hospital number (code known by the principle investigator), while the anonymity status of the patients to any third party was preserved and guaranteed during and after the study.

## Results

### Clinical and demographic data

Out of 183 non-duplicate Enterobacteriaceae isolates from blood cultures, 91 isolates (from 84 BSI episodes in 83 patients) were cefotaxime-resistant. These included *E. coli* (*n* = 68), *K. pneumoniae* (*n* = 17), *Enterobacter cloacae* (*n* = 5) and *E. kobei* (*n* = 1). Seven patients had a polymicrobial BSI with two different Enterobacteriaceae isolated.

Among the 83 patients infected with cefotaxime-resistant isolates, 59.0 % were women, with a median age of 47 years (range 17–78 years); they came from 14 different provinces of Cambodia, predominantly the capital region and adjacent southeastern provinces. The main associated co-morbidities were infection with HIV (*n* = 20; 24.1 %), chronic liver disease (*n* = 18; 21.7 %), chronic renal disease (*n* = 12; 14.5 %) and diabetes mellitus (*n* = 13; 15.7 %).

The primary sources of infection could be clearly identified in 56 out of 84 episodes (66.7 %), and were mainly urogenital (*n* = 29; 51.8 %) and intra-abdominal infections (*n* = 18; 32.1 %), besides respiratory tract infections (*n* = 5; 8.9 %) and skin and soft tissue infections (*n* = 4; 7.1 %).

### Phenotypic resistance patterns

Co-resistance to non-beta-lactam antibiotics was frequently found, in particular for ciprofloxacin, SMX-TMP and gentamicin. For instance, the respective resistance rates for these antibiotics in *E. coli* were 92.6 % (63/68), 95.6 % (65/68) and 76.5 % (52/68). Combined resistance to these three antibiotics in addition to cefotaxime resistance occurred overall in 61.5 % (56/91) of the isolates and was particularly frequent in *E. coli* (88.2 %; 60/68). In contrast, we did not observe resistance to meropenem and amikacin, and found low levels of resistance to colistin (4/91 (4.4 %). Of note, colistin resistance was found in 3 out of 6 *Enterobacter* spp. isolates.

### Resistance mechanisms

ESBL with a unique acquired resistance mechanism to extended-spectrum third-generation cephalosporins (or to cefotaxime) was found in 85 of 91 (93.4 %) isolates, plasmidic AmpC (pAmpC) in 2 of 91 (2.2 %) and a combination of ESBL and pAmpC in 4 out of 91 (4.4 %). In line with the phenotypic findings, no carbapenemase-coding genes were present in any of the isolates.

The distribution frequency of the different ESBLs, CTX-M groups and gene variants is shown in Table 1. *bla*_CTX-M_, present in 86 of 88 ESBL-positive isolates (97.7 %), was by far the most common resistance encoding gene. Most CTX-M types belonged to CTX-M-1 group (*n* = 50) or to CTX-M-9 group (*n* = 30); six isolates harboured a combination of genes from CTX-M- group 1 and CTX-M group 9. CTX-M-15 (*n* = 41) and CTX-M-14 (*n* = 21) were the most common types. Plasmidic AmpC included CMY-2-like in four *E. coli* and DHA-1 in two *K. pneumoniae* isolates, respectively. During the study period, the annual proportion of CTX-M among Enterobacteriaceae fluctuated around an average of 47.0 % (86/183), without a significant trend throughout the years (data not shown).

**Table 1:** Resistance mechanisms underlying cefotaxime resistance in 91 Enterobacteriaceae isolates from bloodstream infections (BSI), Cambodia

ESBL (*n* = 88)		TEM	SHV	OXA-1/30	No. of isolates	+ pAmpC	CipSMXGe^f^	*E. coli* ST131
CTX-M-1 group (*n* = 50)^1^								
	CTX-M-15 (*n* = 41)	−	−	+	18	1 (CMY-2-like)	15	2
		+	−	−	6	−	3	−
		+	−	+	9	−	8	1
		+	+	+	5	−	4	−
		−	−	−	2	1 (CMY-2-like)	0	−
		+	+	−	1	−	1	−
	CTX-M-55 (*n* = 7)	+	−	−	6	1 (CMY-2-like)	5	−
		−	−	−	1	−	1	−
	CTX-M-3 (*n* = 2)	+	−	−	1	−	1	−
		+	+	−	1	−	0	−
CTX-M-9 group (*n* = 30)^b^								
	CTX-M-14 (*n* = 21)	+	−	−	8	−	6	4
		−	−	−	8	−	5	7
		+	+	−	3	−	0	−
		−	+	−	1	−	0	−
		−	−	+	1	−	0	−
	CTX-M-27 (*n* = 9)	+	−	−	4	−	2	1
		−	−	−	4	−	1	3
		+	+	−	1	−	0	−
CTX-M-1 + M-9 group (*n* = 6)^c^								
	CTX-M-15 + CTX-M-14 (*n* = 2)	+	+	−	1	1 (DHA)	1	−
		−	−	+	1	−	0	−
	CTX-M-15 + CTX-M-27 (*n* = 3)	−	−	+	1	−	1	−
		−	−	−	1	−	0	−
		+	−	+	1	−	1	−
	CTX-M-3 + CTX-M-27 (*n* = 1)	+	+	−	1	−	0	−
Non-CTX-M ESBL (*n* = 2)^d^		+	+	−	1	−	0	−
		−	+	−	1	−	0	−
Non-ESBL (*n* = 3)^e^		+	−	−	1	1 (CMY-2-like)	0	−
		+	+	+	1	1 (DHA)	0	−
		+	+	−	1	−	0	−

^a^Includes *E. coli* (*n* = 39), *K. pneumoniae* (*n* = 6) and *Enterobacter* spp. (*n* = 5)

^b^Includes *E. coli* (*n* = 25) and *K. pneumoniae* (*n* = 5)

^c^Includes *E. coli* (*n* = 3) and *K. pneumoniae* (*n* = 2) and *Enterobacter* spp. (*n* = 1)

^d^Includes *K. pneumoniae* (*n* = 3)

^e^Includes *E. coli* (*n* = 1) and *K. pneumoniae* (*n* = 1)

^f^ *CipSMXGe* combined resistance to cefotaxime, ciprofloxacin (*Cip*), SMX-TMP (*SMX*) and gentamicin (*Ge*)

The association of genes encoding for various other non-ESBL beta-lactamase (i.e. *bla*_TEM_, *bla*_SHV_ or *bla*_oxa-1/-30_) was frequent. Of note, the presence of OXA-1/-30 was found in 35 of 46 isolates (76.1 %) with CTX-M-15. Co-resistance to the non-beta-lactam antibiotics (i.e. ciprofloxacin, SMX-TMP and gentamicin) was found more frequently in isolates with CTX-M-1 group (39/50; 78.0 %) as compared to those positive for CTX-M-9 group 9 (14/30; 46.7 %, *p* = 0.007).

No significant differences in antibiotic exposure or nosocomial infection rates was observed between patients with isolates carrying CTX-M-1 versus CTX-M-9 group (data not shown).

### Presence of *E. coli* ST131

**Fig. 1:**
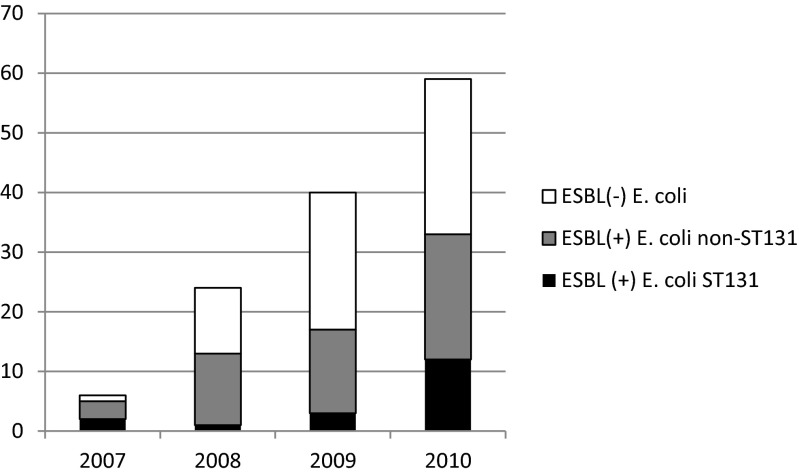
Prevalence of extended-spectrum beta-lactamase (ESBL) *Escherichia coli* ST131 over time (*x*-axis displays calendar years, *y*-axis absolute number of different groups of *E. coli* isolates

Out of 67 CTX-M-positive *E. coli* tested, 18 (26.9 %) were found to be of the B2-ST131-O25B type. Figure 1 displays the evolution of its proportion over time, with a gradual absolute and proportional increase of *E. coli* ST131 being observed over time. Of note, isolates of the ST131 type were more closely associated with CTX-M-9 group (15/18, 83.3 %) as compared to 10 out of 49 (20.4 %) non-ST131 *E. coli*, *p* < 0.001); this was particularly the case for CTX-M-14 (11 out of 18 *E. coli* ST131; 61.1 %). By contrast, CTX-M-1 group genes were significantly more frequent among non-ST131 *E. coli* isolates [36/49 (73.5 %) versus 3/18 (16.7 %) in ST131 *E. coli* isolates, *p* < 0.001].

Besides these associations, infections caused by *E. coli* of the B2-ST131-O25B type were not significantly different from those caused by non-ST131 *E. coli* in terms of patients’ age, gender, co-morbidity and co-resistance to non-beta-lactam antibiotics (Table 2).

**Table 2:** Characteristics of patients infected with CTX-M-positive ST131 *E. coli* as compared to CTX-M-positive non-ST131 *E. coli*

	Infection with *E. coli* ST131		Infection with *E. coli* non-ST131		*p*-Value
	*n* = 18	%	*n* = 49	%	
Female gender	13	72.2	28	57.1	0.397
Age >50 years	7	38.9	23	46.9	0.592
Infection occurred in year 2010	12	66.7	21	42.9	0.104
Infection occurred during wet season^a^	14	77.8	30	61.2	0.255
Nosocomial infection	3	16.7	7	14.3	1.000
Infection with urologic focus	8	44.4	21	42.9	1.000
Died^b^	6	33.3	13	26.5	0.760
Infected with isolate carrying CTX-M-15	3	16.7	32	65.3	**<0.001**
Infected with isolate carrying CTX-M-14	11	61.1	6	12.2	**<0.001**
Underlying diabetes mellitus	2	11.1	10	20.4	0.490
Underlying HIV-infection^c^	3	16.7	10	20.4	1.000
Underlying chronic liver disease	3	16.7	14	28.6	0.527

^a^Wet season: June–November

^b^Outcome assessed at hospital discharge

^c^ *HIV* human immunodeficiency virus

## Discussion and conclusion

In this study, we found that resistance to third-generation cephalosporins among Enterobacteriaceae was widespread in Cambodia and was mostly associated with CTX-M type ESBLs, either alone or in combination with other beta-lactamases, occasionally including pAmpC (i.e. CMY-2-like type in *E. coli* and DHA type in *K. pneumoniae*). We observed a clear predominance of CTX-M-15 and of CTX-M-14, but also a wide variety of genetic patterns, including combinations of two different CTX-M types. The presence of an ESBL was very often associated with co-resistance to ciprofloxacin, SMX-TMP and gentamicin, especially in isolates of CTX-M-15 type.

The overall findings are in line with data from other Asian countries [4]. A surveillance study of 699 invasive Enterobacteriaceae from 11 countries in the Asian-Pacific region [15] and other studies confirmed the high prevalence rates of CTX-M in the region, with predominance of CTX-M-15 on the Indian subcontinent [16], Singapore, Malaysia and the Philippines [17], whereas CTX-M-14 was most frequently found in China [18], South Korea [19] and Taiwan [20]. The presence of non-ESBL mechanisms, e.g. pAmpC (CMY, DHA), also frequently occurs in India, Taiwan, South Korea and Vietnam [15].

Over the years of the study, we also noticed a fast emergence of the ST131 *E. coli* clone and its close association with CTX-M-14. This is in contrast with several reports on the globally emerging, multidrug-resistant CTX-M-15-positive ST131 *E. coli* [3, 21, 22]. However, recent data from Japan also reported the association of the ST131 clone with CTX-M-14 [23]. In China, *E. coli* ST131 was found in association with either CTX-M-14 and CTX-M-15 [18], whereas in neighbouring Laos, *E. coli* ST131 associated with CTX-M-14 is quickly emerging, along with other sequence types, such as ST648 and ST405 [24]. Together with these recent publications, our findings may help to refine the picture of *E. coli* ST131 in Asia, of which the knowledge is still limited [25], its recent epidemiology being probably the result of the vertical (clonal) spread of successful clones and horizontal spread of resistance genes through plasmid transfers within and across different species [26]. Subsequent shuffling and recombination of genes in the same or in different plasmids could explain diversification over time. Also, these findings from invasive isolates are but the tip of the iceberg and a better understanding of the entire *E. coli* epidemiology will require more data on the presence of ST131 in other patient groups, healthy carriers, pet and food animals, and the environment at large.

Finally, in spite of the earlier described association of the ST131 clone with increased clinical severity [3], we found no evidence for this among our patients. The lack of difference in the clinical outcome of infections caused by ST131 *E. coli* has recently been described in a patient series from South Korea [27]. However, the clinical correlate of in vitro observed virulence factors in *E. coli* infections may be very complex [28] and the assessment of their clinical impact requires probably large numbers of more refined patient data with sufficiently long follow-up.

Our study had several limitations. The number of isolates was limited and collected at a single centre over a relatively short period; this may limit their generalisability. Long-term surveillance from multiple sites in Cambodia is needed in order to confirm the trends we observed in our study. Next, given the high rates of comorbidity and of prior antibiotic use in our study population, our results may have overestimated the resistance rates in the community; the rough distinction between ‘community-acquired’ and ‘nosocomial’ isolates did not allow in-depth analysis of these findings. Finally, complete clinical information could not be found for all patients; prospective clinical studies in parallel with surveillance studies are needed in order to assess the clinical context and impact of the newly observed resistance patterns.

Nevertheless, these data represent—to the best of our knowledge—the first detailed description in Cambodia of resistance mechanisms in systematically collected Enterobacteriaceae isolates from BSI.

At a larger scale, our findings highlight the urgent need for an implementation of a nationwide surveillance system in Cambodia [29], whereby particular attention should be given to community-acquired and nosocomial infections alike. The recent introduction of blood culture facilities and laboratory capacity-building in several provincial and public hospitals may constitute a first step towards the creation of a network of skilled laboratories, which may then be able to conduct bacterial surveillance studies [29].

The observed complex combinations of resistance genes suggest intense antibiotic pressure, as confirmed by the high rates of reported prior antibiotic use. This also highlights an urgent need for the global surveillance of antibiotic usage in humans and animals in Cambodia [30], in order to curb the further increase of resistance.
